# Assessment of drivers of spatial genetic variation of a ground‐dwelling bird species and its implications for conservation

**DOI:** 10.1002/ece3.8460

**Published:** 2021-12-20

**Authors:** Florian Kunz, Peter Klinga, Marcia Sittenthaler, Martin Schebeck, Christian Stauffer, Veronika Grünschachner‐Berger, Klaus Hackländer, Ursula Nopp‐Mayr

**Affiliations:** ^1^ Department of Integrative Biology and Biodiversity Research Institute of Wildlife Biology and Game Management University of Natural Resources and Life Sciences, Vienna Vienna Austria; ^2^ Faculty of Forestry Technical University in Zvolen Zvolen Slovakia; ^3^ DIANA ‐ Carpathian Wildlife Research Banská Bystrica Slovakia; ^4^ Central Research Laboratories Natural History Museum Vienna Vienna Austria; ^5^ Department of Forest and Soil Sciences Institute of Forest Entomology, Forest Pathology and Forest Protection University of Natural Resources and Life Sciences, Vienna Vienna Austria; ^6^ Office for Wildlife Biology and Management Mariazell Austria; ^7^ German Wildlife Foundation Hamburg Germany

**Keywords:** conservation genetics, ecological niche modeling, isolation by distance, isolation by resistance, *Lyrurus tetrix*, maximum likelihood population effects (MLPE) models

## Abstract

In modern wildlife ecology, spatial population genetic methods are becoming increasingly applied. Especially for animal species in fragmented landscapes, preservation of gene flow becomes a high priority target in order to restore genetic diversity and prevent local extinction. Within Central Europe, the Alps represent the core distribution area of the black grouse, *Lyrurus tetrix*. At its easternmost Alpine range, events of subpopulation extinction have already been documented in the past decades. Molecular data combined with spatial analyses can help to assess landscape effects on genetic variation and therefore can be informative for conservation management. Here, we addressed whether the genetic pattern of the easternmost Alpine black grouse metapopulation system is driven by isolation by distance or isolation by resistance. Correlative ecological niche modeling was used to assess geographic distances and landscape resistances. We then applied regression‐based approaches combined with population genetic analyses based on microsatellite data to disentangle effects of isolation by distance and isolation by resistance among individuals and subpopulations. Although population genetic analyses revealed overall low levels of genetic differentiation, the ecological niche modeling showed subpopulations to be clearly delimited by habitat structures. Spatial genetic variation could be attributed to effects of isolation by distance among individuals and isolation by resistance among subpopulations, yet unknown effects might factor in. The easternmost subpopulation was the most differentiated, and at the same time, immigration was not detected; hence, its long‐term survival might be threatened. Our study provides valuable insights into the spatial genetic variation of this small‐scale metapopulation system of Alpine black grouse.

## Graphical abstract

By using correlative niche modeling and population genetic methods, we found the spatial genetic structure of Alpine black grouse to be driven by effects of isolation by distance and isolation by resistance.

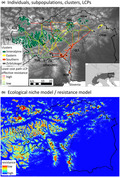

## 1 INTRODUCTION

Spatial population genetic methods are increasingly used in modern wildlife ecology and conservation. Particularly for species in fragmented landscapes, maintaining gene flow is of high relevance to preserve genetic diversity and minimize extinction risks of populations and species (Frankham et al., 2010). Various frameworks and concepts can be applied to identify the spatial distribution of genetic data (Sexton et al., 2014; Wagner & Fortin, 2013; Wang & Bradburd, 2014) and are the basis to understand the structure of populations and infer management strategies. Isolation by distance (IBD) describes the positive relationship between genetic differentiation and geographic distance (usually driven by a species' dispersal; Wright, 1943), a pattern commonly observed in panmictic populations (Sexton et al., 2014). However, the spatial genetic structure of wildlife species can be affected by several co‐occurring factors and processes beyond Euclidean distances (Balkenhol et al., 2016). Therefore, the concept of isolation by resistance (IBR) is of particular interest in wildlife conservation genetics (McRae, 2006). IBR describes the relationship between genetic differentiation and landscape resistance and can be affected by various factors hindering the chance of migration and dispersal through the environment (Wagner & Fortin, 2013; Wang & Bradburd, 2014). Apart from intrinsic, species‐specific drivers such as dispersal strategies (Corrales & Höglund, 2012; Lampert et al., 2003) or dispersal capabilities (Bech et al., 2009), extrinsic factors like landscape topography, vegetational cover, and anthropogenic factors might shape the extent of gene flow and spatial genetic variation (Cushman, 2006). In order to maintain gene flow, preservation and reestablishment of connectivity are primary targets in wildlife conservation (Kettunen et al., 2007). It is thereby essential for conservation management to understand the drivers of spatial genetic variation, especially for connectivity assessments and conservation strategies for ground‐dwelling, elusive species.

Forest grouse (Galliformes, Tetraoninae) are such species. Many populations of these birds are of high conservation concern due to declining trends and increasing habitat fragmentation (Storch, 2007). Well‐documented dispersal capabilities combined with general site fidelity of adult individuals result in genetic structure on a fine spatial scale (Klinga et al., 2015; Rutkowski et al., 2017; Sittenthaler et al., 2018), making grouse important model systems to study drivers of spatial genetic variation. The black grouse (*Lyrurus tetrix*) was specifically targeted by several genetic studies as it is of high conservation concern (Corrales et al., 2014; Höglund, 2009; Rutkowski et al., 2018). Having a distribution range from Great Britain to Siberia, it shows a worldwide decreasing population trend (BirdLife International, 2016), and especially European populations declined dramatically or became extinct in the past decades (Höglund et al., 2007; Larsson et al., 2008; Rutkowski et al., 2018; Segelbacher et al., 2014; Watson & Moss, 2008). Most of the remaining populations are either isolated or exist within a metapopulation context (Caizergues et al., 2003; Höglund et al., 2007). It is consequently listed in Annex I and II of the EU Birds Directive (Directive 2009/147/EC), and special conservation measurements must be taken to ensure its long‐term survival. Core areas of the black grouse Central European distribution are located in the Alps (BirdLife International, 2016; Klaus et al., 1990), where the species shows a strong affinity to the tree‐line ecotone (Sachser et al., 2017). This ecosystem is mainly characterized by a patchy mixture of open, grassy vegetation and woody plants with varying but typically low canopy closure. Alpine black grouse usually avoid patches with a dense tree canopy closure (Immitzer et al., 2014; Patthey et al., 2012; Sachser et al., 2017; Schweiger et al., 2012), and open, elevated habitat patches are preferred sites for lekking. Dispersal of black grouse is typically sex‐biased with natal dispersal of females and philopatry of males (Caizergues & Ellison, 2002; Corrales & Höglund, 2012). Female dispersal usually occurs over distances of up to 8 km (Caizergues & Ellison, 2002; Marjakangas & Kiviniemi, 2005; Warren & Baines, 2002; Willebrand, 1988). Although in rare events, black grouse traverse longer distances in flight (potentially enabling gene flow over impermeable landscapes), it is in general a sedentary bird species, responding sensitively to the spatial structure of habitats. Being mainly ground‐dwelling (Klaus et al., 1990), black grouse therefore serves as an indicator species for its ecosystem (Storch, 2007), and habitat factors are assumed to be key factors for movement behavior and dispersal.

**FIGURE 1:**
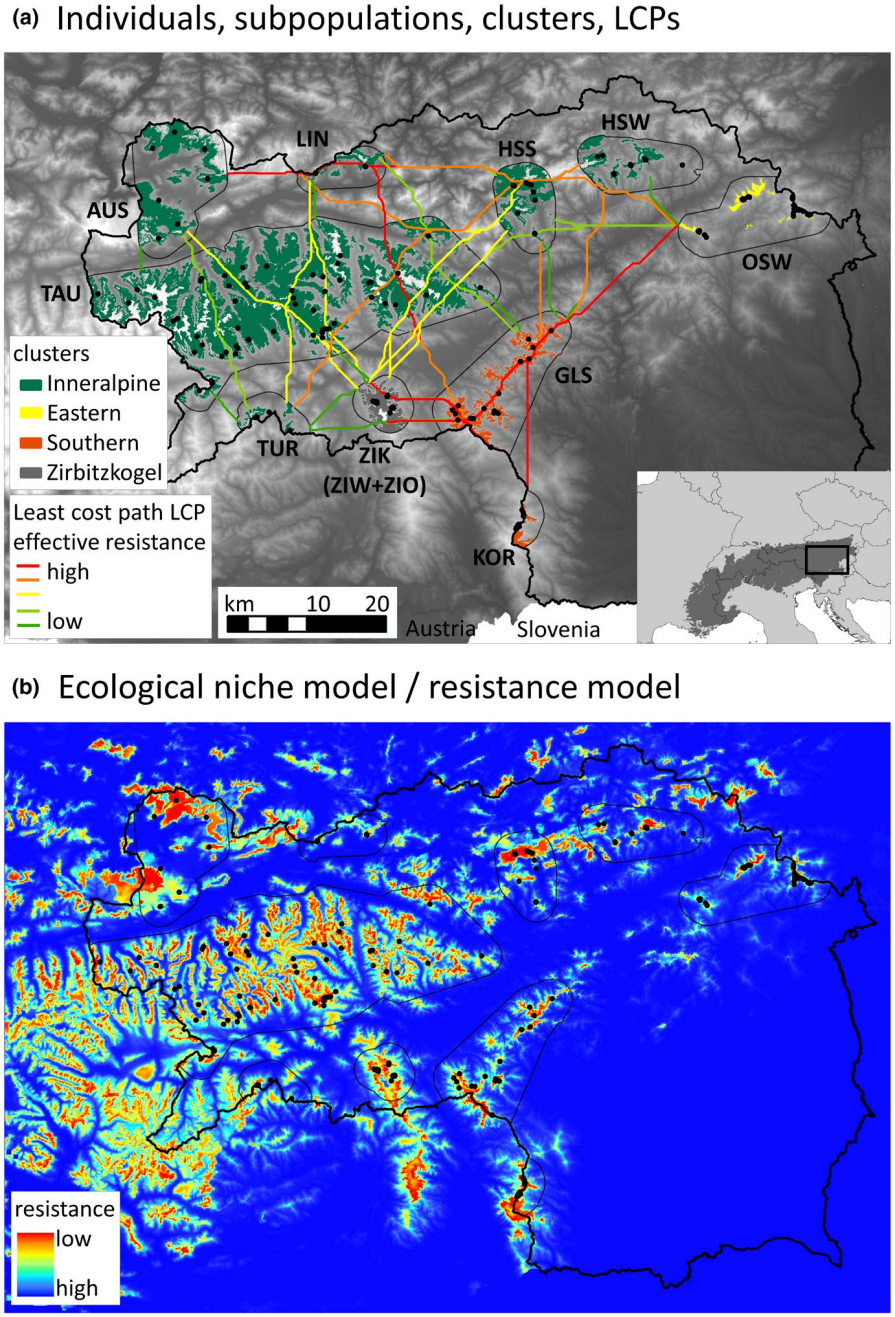
Results of population genetic analyses, ecological niche modeling, and landscape genetic approaches on 195 Styrian black grouse individuals. (a) Digital elevation model of the study area Styria, with all 195 individuals, classified in 10 subpopulations (black outline, 5‐km buffer around presence points, identified by Sittenthaler et al., 2018) and four clusters (green‐, yellow‐, orange‐, and gray‐colored areas of suitable habitat, as identified in this study). Least‐cost‐paths by Linkage Mapper 1.1 were classified into five quantiles of effective resistances calculated by Circuitscape 4.0. The inset shows the area of the Alps (dark gray) provided by the European Environment Agency and the location of our study area (black square). (b) Ecological niche model by MaxEnt 3.4.1, representing the resistance surface

Black grouse habitats within the Alps are naturally separated by high mountain ridges and low valleys (Caizergues & Ellison, 2002). Over the last decades, abandonment of alpine pastures (Groier, 2010) and impacts of climate change affected the plant community distribution (Gehrig‐Fasel et al., 2007; Theurillat & Guisan, 2001), which resulted in a distinct loss of open habitats and in altitudinal shifts of the tree‐line ecotone (Tasser et al., 2007), significantly reducing the available habitat for black grouse. Furthermore, habitats became increasingly fragmented by human settlements, agricultural areas, expanding skiing areas, wind power facilities, and other human activities (Arlettaz et al., 2007; Coppes et al., 2017; Immitzer et al., 2014; Ingold, 2005). These effects become particularly important at the marginal areas of the species' distribution. For the easternmost black grouse occurrences of the Alpine distribution, situated in the Austrian province of Styria (Figure 1), genetic differentiation into distinct clusters has already been observed (Sittenthaler et al., 2018), and multiple extinction events of marginal subpopulations have been documented in the past decades (Wöss & Zeiler, 2003). It remained unclear whether the spatial genetic variation was driven by the mere geographic distance or the resistance of the landscape. Yet, such knowledge is of major importance to infer targeted conservation actions, in order to preserve threatened populations and to adjust ongoing landscape planning processes.

Here, we aimed to study the population genetic structure and habitat suitability of a Central European black grouse metapopulation system to infer drivers of spatial genetic variation and to understand their effects on the conservation status of the species. We modeled connectivity using least‐cost‐path (LCP) lengths and effective resistances and parameterized regression‐based landscape genetic analyses among individuals and subpopulations. Our study helps to understand drivers of the genetic structure of Alpine black grouse populations at the edge of their range. This is the basis to infer conservation strategies and can help to prevent the loss of this characteristic bird species of Alpine ecosystems.

## 2 METHODS

### 2.1 Study site and collection of samples

Samples of black grouse were obtained from the entire Austrian province of Styria (Figure 1), representing the easternmost occurrence of the species' Alpine distribution range (BirdLife International, 2016). The study area shows a high portion of mountain areas, ranging from 200 to almost 3000 m.a.s.l., a high cover of conifer forests (>55%), a prominent portion of alpine meadows (7%), and gradients between alpine and pannonic climate (Land Steiermark, 2019). Black grouse occurrences are structured in subpopulations based on topographical criteria and average dispersal distances (Sittenthaler et al., 2018). Several subpopulations at the edges of the distribution range have already gone extinct (Wöss & Zeiler, 2003), and the remaining 10 subpopulations form a metapopulation system (Table 1; Sittenthaler et al., 2018). We used genetic data of black grouse individuals from a previous population genetic survey from all subpopulations (Sittenthaler et al., 2018). Samples were obtained from feces, feathers, and muscle tissue (*n* = 250) and stored in ethanol (for muscle tissue) and frozen (for feces and feathers). DNA extraction and polymerase chain reaction (PCR) amplification were performed as described in Sittenthaler et al. (2018). Each individual was genotyped at nine microsatellite loci using a multiple tubes approach for noninvasive samples (Navidi et al., 1992; Taberlet et al., 1996). A total of 195 individuals were fully genotyped. A consensus genotype was accepted when at least two (for heterozygote loci) or three (for homozygote loci) independent replications of a single allele were recorded.

**TABLE 1:** Characterization of the subpopulations within the metapopulation system of black grouse in Styria

Subpopulation	Abbreviation	*P*_E_	*N*	cluster assignment	*H*_O_	*H*_E_	*F*_IS_
Aussee	AUS	1200	7	Inneralpine	0.65	0.66	0.02
Liezen North	LIN	450	5	Inneralpine	0.69	0.63	−0.09
Hoschschwab South	HSS	925	13	Inneralpine	0.76	0.71	−0.07
Hochschwab West	HSW	925	13	Inneralpine	0.68	0.70	0.02
Tauern	TAU	6.850	56	Inneralpine	0.66	0.69	0.04
East Styria/Wechsel	OSW	400	41	Eastern	0.60	0.64	0.07
Turrach	TUR	850	4	Inneralpine	0.75	0.69	−0.08
Zirbitzkogel	ZIK	500	18	Zirbitzkogel	0.54	0.62	0.12
Gleinalm/Stubalm	GLS	700	23	Southern	0.62	0.68	0.09
Koralm	KOR	150	15	Southern	0.61	0.62	0.03

Note

Overall *F*_IS_: −0.04; Overall *F*_IT_: 0.04; Overall $F_{\text{ST}}$: 0.08.

Cluster assignment based on Sittenthaler et al. (2018), results from *memgene* and indices of fixation and differentiation. Population size estimates are rough expert‐based estimates to characterize the subpopulations.

Abbreviations: *F*_IS_, inbreeding coefficient; *H*_E_, expected heterozygosity; *H*_O_, observed heterozygosity; *N*, number of individual genotypes; *P*_E_, population size estimate (Sittenthaler et al., 2018).

### 2.2 Population genetic analysis

Summary statistics were calculated per subpopulation using the R package *hierfstat* 0.5‐7 (Goudet, 2005). In addition to $F_{\text{ST}}$ values (Weir & Cockerham, 1984), we calculated the pairwise fixation indices $G_{\text{ST}}$ (Nei & Chesser, 1983) and $G_{\text{ST}}^{\prime }$ (Hedrick, 2005) and the differentiation index $D_{\text{Jost}}$ (Jost, 2008), using the R package *diveRsity* 1.9.9 (Keenan et al., 2013). As $G_{\text{ST}}^{\prime \prime }$ (Meirmans & Hedrick, 2011) is not implemented within *diveRsity*, we used the R package *mmod* 1.3.3 (Winter, 2012) and calculated bias‐corrected confidence intervals following the method implemented in *diveRsity*. For all indices, confidence intervals were based on 10,000 bootstrap iterations. Although correlated (Pearson's correlation coefficients ranging from 0.8 to 0.9), these indices quantify complementary aspects of population structure and should therefore be considered separately for subsequent analyses (Jost et al., 2018; Meirmans & Hedrick, 2011). To assess clustering within the genetic dataset, a principle component analysis (PCA) was calculated using the R package *adegenet* 2.0.1 (Jombart, 2008; Jombart & Ahmed, 2011) in addition to the discriminant analysis of principle components (DAPC) and Structure analyses by Sittenthaler et al. (2018). Given the previously reported low amount of genetic differentiation among subpopulations (Sittenthaler et al., 2018), we further used the R package *memgene* 1.0.1 (Galpern et al., 2014) to explore spatial genetic patterns in detail. *memgene* was specifically designed to detect and visualize weak or cryptic structure within a genetic pattern by using Moran's eigenvector maps (MEMs; Galpern et al., 2014), thus being a suitable approach to detect genetic structure in our study system. We used the function mgQuick to assess population structure, with the response variable being the proportions of shared alleles *D*_PS_ (calculated with *memgene*) among individuals. Subpopulations in our study area were assigned to clusters based on the combined interpretation of Structure and DAPC results by Sittenthaler et al. (2018), our *memgene* analysis and significant indices of genetic fixation and differentiation.

Furthermore, we estimated recent migration rates to analyze potential asymmetric migration using BayesAss 3.0.4 (Wilson & Rannala, 2003). Migration rates were calculated between clusters based on the analyses of population genetic structure (Table 2). We conducted 10 independent repeats of 50 ∗ 10^6^ iterations (including 5 ∗ 10^6^ iterations burn‐in) with a sampling frequency of 2000, each initiated with a different random seed for each dataset. In order to keep the acceptance rates for proposed changes between 40% and 60%, delta values were adjusted to Δ*m* = 0.1, Δ*a* = 0.3, and Δ*f* = 0.7. Convergence of chains was confirmed using Tracer 1.7.1 (Rambaut et al., 2018) and by checking for concordance between repeats. We used the Bayesian deviance as calculated by Meirmans (2014) in R 3.6.0 (R Core Team, 2019) to search for the best fitting model (the one with the lowest Bayesian deviance was selected) (Faubet et al., 2007). Credible intervals (CIs 95%) of migration rates were calculated as standard deviation multiplied by 1.96 as described in the program's manual. Migration rates that included zero within their 95% CI were considered not significant.

**TABLE 2:** Migration rates as estimated by BayesAss 3.0.4 with 95% credible intervals among the genetic clusters of black grouse as in Table 1

To	From			
	Inneralpine	Eastern	Southern	Zirbitzkogel
Inneralpine	**0.700** (±0.040)	0.046 (±0.100)	**0.251** (±0.112)	0.006 (±0.012)
Eastern	0.021 (±0.040)	**0.812** (±0.248)	0.154 (±0.258)	0.013 (±0.025)
Southern	0.030 (±0.044)	0.019 (±0.037)	**0.941** (±0.057)	0.010 (±0.020)
Zirbitzkogel	0.018 (±0.034)	0.056 (±0.133)	**0.239** (±0.122)	**0.687** (±0.040)

Note

Significant values based on the credible intervals are in bold emphasis.

### 2.3 Ecological niche modeling and resistance surface

In order to parameterize a model representing the resistance of the landscape to movement and dispersal for black grouse, we used a correlative ecological niche model (ENM). The process of parameterization of resistance models is broadly discussed (Mateo‐Sánchez et al., 2015; Milanesi, Holderegger, Caniglia, et al., 2017; Roffler et al., 2016; Wang et al., 2008), and several approaches have been suggested. Black grouse are mainly ground‐dwelling and react sensitively to habitat structures. Movement and dispersal are most probably directly linked to habitat factors, as suitable habitats provide food resources and protection (against predators and adverse weather conditions). Therefore, we assume that the resistance of a landscape to movement and dispersal is best reflected by the distribution of suitable habitat areas (Milanesi, Holderegger, Caniglia, et al., 2017). Furthermore, ENMs have already been used successfully to parameterize resistance surfaces for the closely related Western capercaillie (*Tetrao urogallus*) (Milanesi et al., 2017). Accordingly, we selected 15 topographic, climatic, and land cover variables that might affect dispersal and movement (Table 3).

**TABLE 3:** Environmental input data used for the ecological niche modeling of black grouse in Styria with MaxEnt 3.4.1 (Phillips et al., 2006; Phillips & Dudík, 2008)

Environmental variable	Final model contribution (%)	Source
Distance to subalpine grasslands	55.7	Derived from the land use classification
Altitude	37.8	Derived from a digital elevation model (DEM) by LiDAR data (Land Kärnten, 2015)
Land use classification	4.3	Classified into eight categories based on Wrbka et al. (2002)
Distance to human settlements and industrial areas	1.6	Derived from the land use classification
Ruggedness, vector ruggedness measure (VRM)	0.7	Derived from the DEM following Sappington et al. (2007), neighborhood size: 11
Aspect	—	Derived from the DEM
Slope	—	Derived from the DEM
Buffered single tree individuals above 1200 m.a.s.l.	—	Derived from LiDAR data (GIS‐Steiermark, 2018), includes vegetation between 6 and 15 m height outside of areas classified as forest
Distance to single tree individuals	—	Derived from the single tree individuals
Climatic variables (duration of vegetation period, precipitation per season, days of frost, and days of snow cover)	—	Klimaatlas Steiermark/climate data (GIS‐Steiermark, 2018)
Tree composition	—	Waldatlas Steiermark/forest data (GIS‐Steiermark, 2018)
Tree height	—	Waldatlas Steiermark/forest data (GIS‐Steiermark, 2018)

Note

Final model contribution gives the relative contribution of the variable to the final model. Most important variable based on jackknife tests was altitude.

The topographic variables (altitude, slope, exposure, and ruggedness, Sappington et al., 2007) were calculated based on the digital elevation model. The climatic variables were taken from the official geodata catalog of climate of the province of Styria (GIS‐Steiermark, 2018). The land cover dataset was based on an extensive land cover classification (Wrbka et al., 2002). It comprises 42 landscape types, which were grouped into the eight categories relevant for black grouse (Table 4): summits and glaciers; subalpine grasslands (including pastures and meadows); continuous forests; lowland forest patches; submountainous grasslands; lowland grasslands and pastures; lowland arable land; and human settlements and industrial areas. We included two variables representing the distance to the land cover type positively (subalpine grasslands) or negatively (human settlements and industrial areas) affecting black grouse distribution. Hence, we accounted for potential push or pull effects of these areas. As the inclusion of local habitat structures is crucial for ENMs to parameterize resistance surfaces (Milanesi, Holderegger, Bollmann, et al., 2017), we included variables representing tree height, tree composition, and the existence of single tree individuals. Tree height and composition directly link to black grouse habitat preferences and were based on the official geodata catalog of forestry of the province of Styria (GIS‐Steiermark, 2018). Single trees in open subalpine areas might also positively affect habitat suitability by offering resting sites and food resources. We therefore generated a dataset representing single trees in subalpine areas by extracting vegetation between 6 and 15 m height outside of the land cover categories “continuous forest” and “lowland forest patches” from light detection and ranging (LiDAR) data (GIS‐Steiermark, 2018). Additionally, we calculated distance to single trees to assess potential pull effects. Based on Pearson's correlation coefficient, we excluded highly correlated variables (coefficients ≥ |0.7|). For all data, we used a resolution of 100 m grain size. Preparation steps and further spatial analyses were done in ArcGIS 10.5 (ESRI, 2016). The study area was buffered 20 km around the political boundary of Styria, allowing the analyses to explore areas of biological relevance beyond administrative borders.

**TABLE 4:** Summary of land use classification by Wrbka et al. (2002) into eight categories relevant for black grouse in Styria used in the present study

Land use category used in the present study	Land cover (%)	Identifier of Wrbka et al. (2002)
Summits and glaciers	3.2	101
Subalpine grasslands and pastures	7	102, 103
Continuous forests	22.8	201
Lowland forest patches	35.3	202, 203, 204, 205
Submountainous grasslands and pastures	8.6	301, 302, 303
Lowland grasslands and pastures	11.3	304, 305, 307, 312, 313
Lowland arable land	9.9	401, 402, 404, 405, 406, 407, 411, 604
Human settlements and industrial areas	1.9	701, 702, 703, 704, 705, 706

Note

Land cover displays the proportion of study area covered by the respective category.

The ENM was calculated using maximum entropy modeling implemented in MaxEnt 3.4.1 (Phillips et al., 2006; Phillips & Dudík, 2008). The underlying principle of maximum entropy uses machine learning concepts to minimize the difference between two probability density functions of environmental variables, one based on our presence locations and the other one based on the entire study area (background locations) (Elith et al., 2011). We calibrated models with varying sets of environmental variables and regularization parameters and combinations of features (Merow et al., 2013; Phillips et al., 2017). We followed a stepwise top‐down procedure of model selection, evaluating model fit and adequacy by their average area under the receiver operating characteristics curve (AUC) value through cross‐validation and together with regional experts as recommended by Morales et al. (2017). The final model parameters were set to 20 replications of 5,000 iterations, and the regularization parameter was set to 1.5. To account for a potential sampling bias, we included background manipulation via a Gaussian kernel density of sampling locations calculated with SDMtoolbox 2.2 (Brown, 2014; Brown et al., 2017) as bias file. The final ENM was inverted into a resistance surface using SDMtoolbox 2.2. Additionally, we created an alternative resistance surface based on an inverted ENM of altitude only (altitude_inv), as altitude was the most explanatory variable beside land cover classification in the ENM.

### 2.4 Measures of IBR

We applied two distinctly different approaches to extract distances and resistance values of the resistance surface that might explain IBR: (1) LCP lengths were extracted according to the cost distance approach (Adriaensen et al., 2003), and (2) effective resistances were calculated according to the circuit theory approach (McRae et al., 2008). Whereas the cost distance approach assumes an individual's full a priori knowledge of the landscape when calculating the LCP, circuit theory assumes random movement and therefore yields higher connectivity where higher redundancy in travel routes exist (McClure et al., 2016). LCPs and effective resistances between subpopulations (areas defined as suitable habitat within a conservative 5 km buffer around individual presence points; Figure 1) were generated using the geographical information system routine within LinkageMapper 1.1 (McRae & Kavanagh, 2011) and PinchPoint Mapper (McRae, 2012) (making use of Circuitscape 4.0; McRae et al., 2013).

### 2.5 Identifying spatial genetic pattern

At the individual level, we used the function mgLandscape within *memgene* to address whether IBD or IBR might explain the spatial genetic pattern. This function computes LCPs from provided resistance surfaces to extract MEM eigenvectors and subsequently performs a regression framework. We used *D*_PS_ as response variable and the following landscape distances as predictors (Table 5): Euclidean distances resembling IBD (Euc. dist.), our resistance surface based on the ENM resembling IBR (res. surface), and the resistance surface based on altitude alone (altitude_inv). By including altitude as a predictor, we assessed whether IBR effects are driven by the complex ENM (including topography, climate, and land cover) or by altitude alone (irrespective of anthropogenic influence).

**TABLE 5:** Comparison of the proportion of spatial genetic variation ($R_{\text{adj}}^{2}$) among black grouse individuals in Styria explained by Moran's eigenvector maps derived from different models

Model	[abc]	*P*[abc]	[a]	*P*[a]	[c]	*P*[c]	[b]	[d]
Euc. dist.	0.080	0.001	0.052	0.001	0.005	0.060	0.023	0.920
res. surface	0.074	0.001	0.047	0.001	0.003	0.126	0.024	0.926
altitude_inv	0.055	0.001	0.028	0.001	−0.001	0.631	0.029	0.945

Note

The table describes the proportion of variation in pairwise genetic distances that can be attributed to the different spatial predictors [abc] and to the particular pattern in the landscape resistance surface [a], the coordinates of the individuals in the landscape resistance surface [c], or to confounded pattern of the landscape resistance surface and coordinates [b]. Additionally, residuals not explained by spatial predictors are reported [d]. *P*[abc], *P*[a], and P[c] represent the *p* values of each calculated proportion. Tested models are Euclidean distances (Euc. dist.), pairwise least‐cost‐path (LCP) lengths between individuals across the resistance surface based on the ENM (res. surface), and pairwise LCPs between individuals across a resistance surface based on altitude only (altitude_inv).

At the subpopulation level, we contrasted IBD versus IBR using the regression framework within the function mgLandscape_list by Polato et al. (2017). Although following the same approach as mgLandscape within *memgene*, this adapted function allowed us to test the aforementioned indices of pairwise genetic fixation and differentiation as response variables against pairwise geographic distances as predictors. The pairwise Euclidean distances (IBD), LCP lengths (IBR), and effective resistances (IBR) were used as predictors. Additionally, we calculated maximum likelihood population effects (MLPE) models (Clarke et al., 2002) implemented in the R package *ResistanceGA* (Peterman, 2018). MLPE models account for nonindependence of pairwise distance data due to population effects and have been identified as best‐suited regression‐based approaches for model selection (Shirk et al., 2017, 2018). We used the same response and explanatory variables as for the mgLandscape_list approach. Due to strong correlations between the explanatory variables and the small sample size within each model, each variable was tested separately resulting in 20 models (five response variables and three explanatory variables plus a null model assuming that the response variable is constant for the explanatory variable). We then applied deltas and weights of the Akaike Information Criterion corrected for small sample sizes (AICc; Anderson & Burnham, 2002) and *R* ^2^ to compare the candidate models and select the best model (Row et al., 2017).

## 3 RESULTS

### 3.1 Spatial genetic structure

**FIGURE 2:**
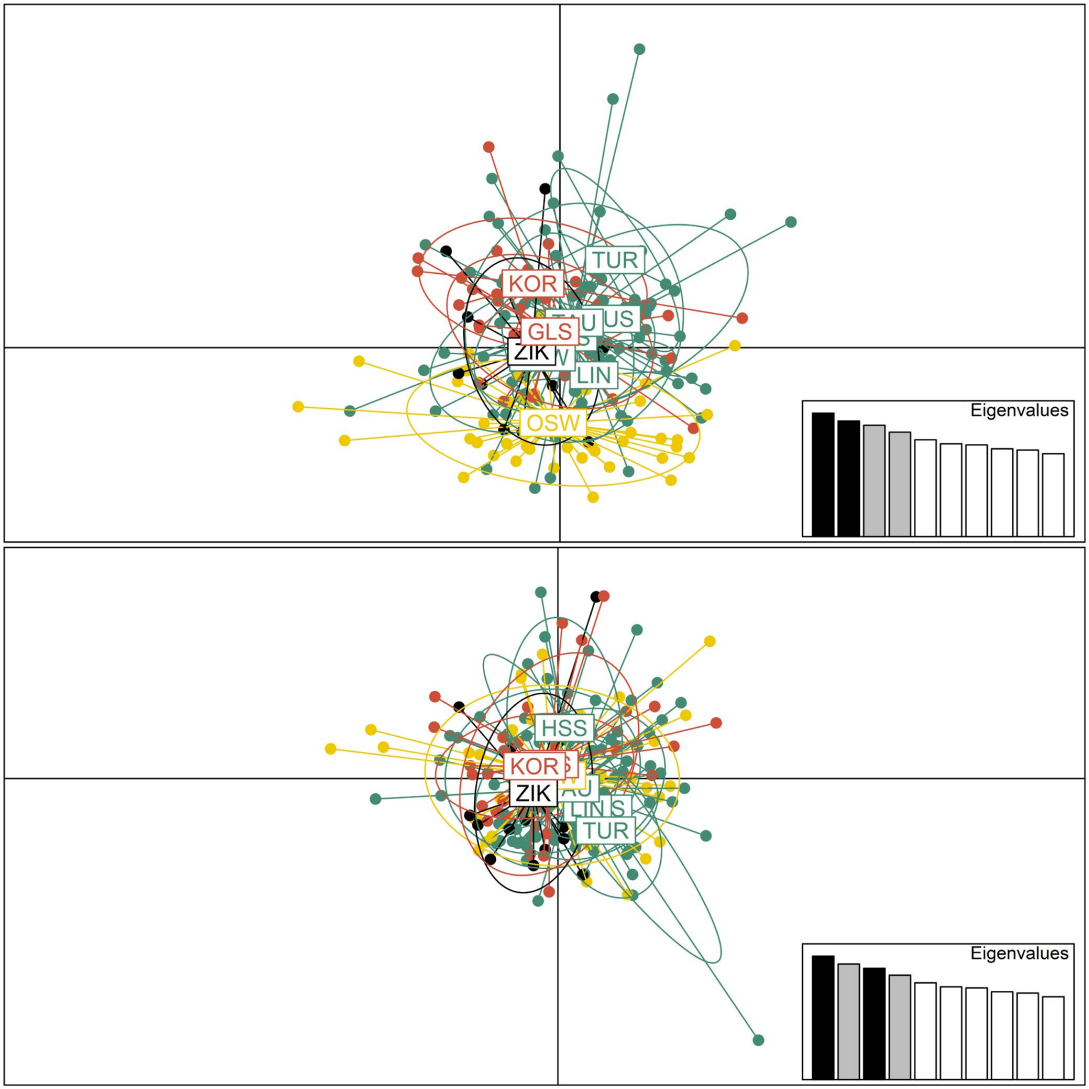
Principal component analysis with four retained PCs of the 195 Styrian black grouse genotypes. PC1 (*x* axis; 3.9% explained variance) versus PC2 (*y* axis; 3.6% explained variance) (top) and PC1 (*x* axis, 3.9%) versus PC3 (*y* axis, 3.5%) (bottom). Different colors indicate the assignment of subpopulations to four clusters

**FIGURE 3:**
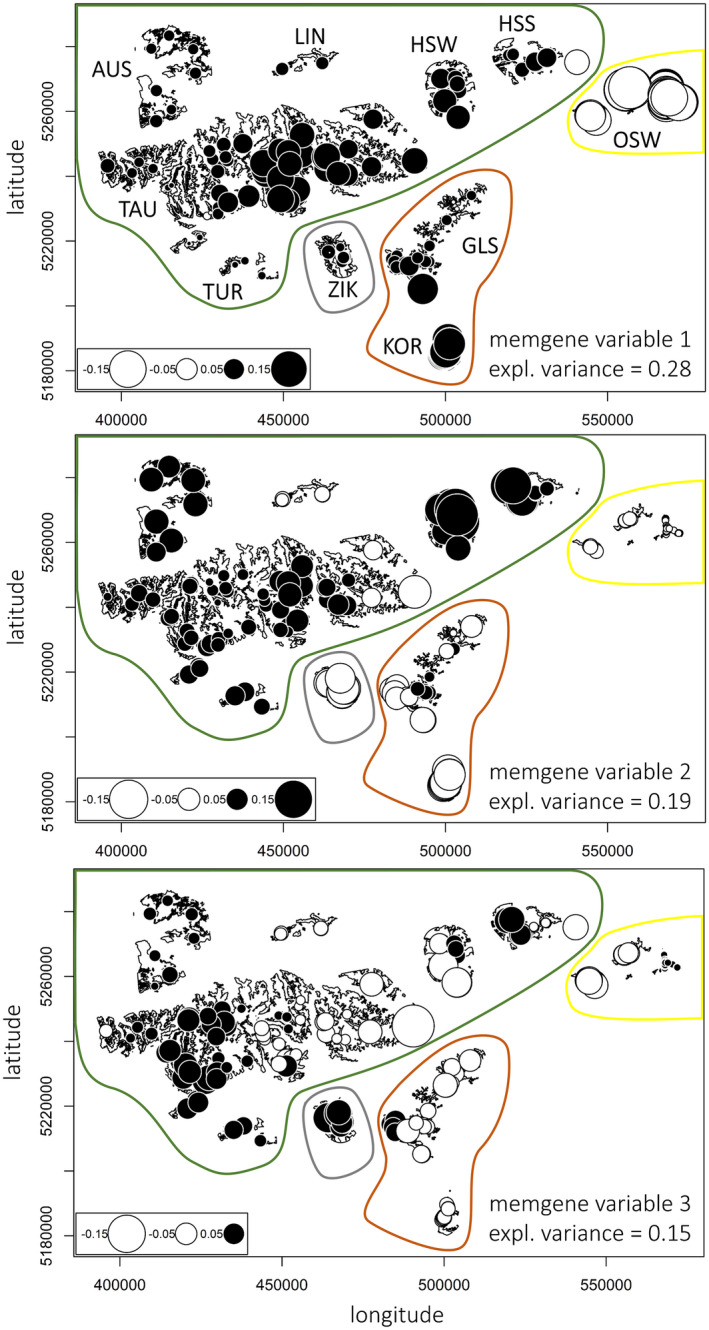
Spatial genetic structure of the 195 Styrian black grouse samples as found by *memgene* 1.0.1 (Galpern et al., 2014). Circles of similar size and color indicate individuals with similar scores (large black and large white circles describe opposite extremes). The first *memgene* variable explains 28% of the spatial genetic variation and the second and third variable 19% and 15%, respectively. Colored polygons indicate the assignment of subpopulations to the four clusters. Axes in UTM WGS84

Overall, a low amount of genetic differentiation among subpopulations was detected. Although the PCA could not resolve a clear cluster assignment (Figure 2), the spatial genetic structure detected by the mgQuick approach of *memgene* (Figure 3) indicated the presence of clusters. The first *memgene* variable explaining the highest amount of spatial genetic variation found the subpopulation OSW to be distinct. The second variable suggested a cluster of the northern subpopulations, and subpopulation ZIK seemed to be connected to the southeastern ones. The third variable showed mixed results for the northern subpopulations, and ZIK seemed to be distinct from the southeastern subpopulations. Significant indices of genetic fixation and differentiation (Table 6 and Table A1) provided further evidence for genetically discrete clusters; both OSW and ZIK were differentiated. Taken together, our data and the Structure and DAPC results by Sittenthaler et al. (2018) imply that the ten subpopulations can be differentiated into four clusters (Table 1). The subpopulations AUS, LIN, HSS, HSW, TAU, and TUR are situated in the Central Alps and together formed the Inneralpine cluster. The southern subpopulations GLS and KOR formed a cluster called Southern. The easternmost subpopulation OSW was the most differentiated and formed its own cluster Eastern. The subpopulation ZIK showed ambiguous results and was therefore assigned its own cluster Zirbitzkogel.

**TABLE 6:** Pairwise $F_{\text{ST}}$ (Weir & Cockerham, 1984) and $G_{\text{ST}}^{\prime \prime }$ (Meirmans & Hedrick, 2011) comparisons among black grouse subpopulations in Styria

Subpopulation ID	AUS	LIN	HSS	HSW	TAU	OSW	TUR	ZIK	GLS	KOR
AUS	—	0.044	0.044	0.073	0.008	**0.119**	0.040	**0.121**	0.034	0.091
LIN	0.017	—	0.070	0.116	0.026	0.081	0.156	**0.132**	0.120	**0.221**
HSS	0.016	0.026	—	0.047	0.040	**0.121**	**0.198**	**0.223**	0.040	**0.162**
HSW	0.022	0.037	0.015	—	0.069	**0.124**	0.136	**0.144**	0.039	**0.171**
TAU	0.001	0.005	0.012	**0.021**	—	**0.093**	0.079	**0.116**	0.037	**0.100**
OSW	0.039	0.026	**0.040**	**0.041**	**0.030**	—	**0.306**	**0.141**	**0.085**	**0.203**
TUR	0.015	0.063	**0.064**	0.041	0.023	**0.103**	—	**0.273**	**0.190**	**0.242**
ZIK	0.040	0.045	**0.076**	**0.048**	**0.038**	**0.050**	**0.094**	—	**0.123**	**0.177**
GLS	0.007	0.035	0.012	0.010	0.011	**0.028**	**0.056**	**0.040**	—	0.036
KOR	0.032	**0.083**	**0.056**	**0.058**	**0.032**	**0.073**	**0.087**	**0.065**	0.010	—

Note

$F_{\text{ST}}$ values below the diagonal and $G_{\text{ST}}^{\prime \prime }$ above. Significant values based on 95% bias corrected confidence intervals in bold.

The proportion of genetic variation found by *memgene* that can be attributed to spatial patterns ($R_{\text{adj}}^{2}$, Galpern et al., 2014) was 0.07, indicating weak overall genetic structure. This is in line with the indices of fixation and differentiation showing overall low fixation and differentiation (range of values for $F_{\text{ST}}$: 0.001–0.103; $G_{\text{ST}}$: 0.001–0.053; $G_{\text{ST}}^{\prime }$: 0.007–0.207, $G_{\text{ST}}^{\prime \prime }$: 0.008–0.306; $D_{\text{Jost}}$: 0.001–0.124). Results of the first and second *memgene* variables (explaining 28% and 19% of the spatial genetic variation, respectively) resolved the four genetic clusters, with ambiguous assignments for ZIK. In estimation of migration rates, BayesAss chains converged well, and log‐likelihood and Bayesian deviance were comparable between repeats. The estimates indicate unidirectional migration patterns between the clusters (Table 2). Individuals appeared to be migrating from Southern into the Zirbitzkogel and Inneralpine clusters. Migration rates from Southern to Eastern were not significant. Therefore, no immigration into Southern and Eastern was found.

### 3.2 Ecological niche modeling and resistance surfaces

All climatic variables were excluded due to their high correlation with altitude prior to the parameterization of the ENM. The final ENM (Figure 1) comprised the following five environmental variables reflecting relevant topographical and land cover criteria for black grouse (model contributions in parentheses): distance to subalpine grasslands (55.7%); altitude (37.8%); land use classification (4.3%); distance to human settlements and industrial areas (1.6%); and terrain ruggedness (0.7%) (Table 3). The model corresponded well to regional expert assessment and showed an averaged test AUC of 0.954, which indicated strong model fit and high predictive performance. The single most important variable in terms of information not covered by other variables was altitude.

### 3.3 Identifying spatial genetic pattern

Within the mgLandscape approach on the individual level, MEM eigenvectors derived from Euclidean distances ([abc], Table 5) between individuals explained a slightly higher proportion of spatial genetic variation ($R_{\text{adj,[abc]}}^{2}$ = 0.08) than MEM eigenvectors derived from the resistance surface based on the ENM and the resistance surface based on altitude alone ($R_{\text{adj,[abc]}}^{2}$ = 0.074 and 0.055, respectively). The fraction of genetic distance that is explained by the model [a] is notably higher than the fraction explained by coordinates [c], indicating good model fit (Table 5). Although Euclidean distances (testing for the effect of IBD) are therefore preferred over the resistance surface based on the ENM (testing for the effect of IBR), the small difference in the proportions of spatial genetic variation explained by the spatial predictors [abc] suggests that IBD and IBR effects cannot be easily distinguished. Among subpopulations, the mgLandscape_list approach was not able to detect significant Moran's eigenvectors, as the spatial signal within the pairwise genetic distance matrices was presumably too weak. All MLPE models, however, showed positive signs of relationships between the predictors and dependent variables. The predictors were significant (*α* = 0.05) for all models except the ones built with $D_{\text{Jost}}$ as dependent variable. The models using LCP length as predictor were preferred for all indices of fixation or differentiation (Table 7).

**TABLE 7:** Maximum likelihood population‐effects models for the black grouse subpopulations in Styria, ranked by weights (*w*) of the delta of the corrected Akaike Information Criterion for small sample sizes (ΔAICc) and *R* ^2^ (marginal/conditional)

Response variable	Explanatory variable	ΔAICc	*w*	*R* ^2^
$F_{\text{ST}}$ (Weir & Cockerham, 1984)	LCP length	0.00	0.60	0.17/0.66
	Euclidean dist.	0.97	0.37	0.16/0.65
	Effective resist.	6.09	0.03	0.10/0.58
	Null model	8.84	0.01	0.00/0.57
$G_{\text{ST}}$ (Nei & Chesser, 1983)	LCP length	0.00	0.61	0.19/0.66
	Euclidean dist.	1.01	0.37	0.16/0.65
	Effective resist.	6.49	0.02	0.10/0.56
	Null model	9.54	0.01	0.00/0.55
$G_{\text{ST}}^{\prime }$ (Hedrick, 2005)	LCP length	0.00	0.59	0.20/0.64
	Euclidean dist.	0.88	0.38	0.19/0.63
	Effective resist.	6.65	0.02	0.10/0.53
	Null model	9.57	0.00	0.00/0.52
$G_{\text{ST}}^{\prime \prime }$ (Meirmans & Hedrick, 2011)	LCP length	0.00	0.59	0.20/0.64
	Euclidean dist.	0.87	0.38	0.19/0.64
	Effective resist.	6.66	0.02	0.10/0.54
	Null model	9.66	0.00	0.00/0.53
$D_{\text{Jost}}$ (Jost, 2008)	LCP length	0.00	0.28	0.07/0.38
	Euclidean dist.	0.17	0.26	0.06/0.37
	Null model	0.39	0.23	0.00/0.34
	Effective resist.	0.44	0.23	0.06/0.33

Note

Response variables were fixation and differentiation indices of genetic distances; explanatory variables were a null model, Euclidean distances (Euclidean dist.), least‐cost‐path (LCP) lengths based on the ecological niche model (ENM) (LCP length), and effective resistances (Effective resist.).

## 4 DISCUSSION

We applied several consecutive analyses to disentangle the drivers of spatial genetic variation within an Alpine black grouse metapopulation system at the easternmost edge of the species' distribution. Although low levels of population differentiation and only a slight difference among models testing for IBD or IBR were found, our results provide valuable insights into the spatial genetic pattern of this small‐scale metapopulation system with a high conservation concern.

### 4.1 Migration rates and population structure

The migration rates estimated by BayesAss indicated emigrating individuals from the two clusters, Eastern and Southern. However, BayesAss estimates should be viewed with caution as the maximum proportion of immigrated individuals within a cluster is assumed to not exceed one third of its size (Faubet et al., 2007). Although the overall genetic differentiation is low within our study system, black grouse are sedentary birds with intermediate juvenile dispersal (Caizergues & Ellison, 2002; Marjakangas & Kiviniemi, 2005; Warren & Baines, 2002), presumably not violating this assumption. Additionally, BayesAss decreases in accuracy when sample sizes are differing among subpopulations (Meirmans, 2014). Although this is the case in our study (as is for the most studies on rare and elusive species), BayesAss estimates correspond well to our other results. Especially the subpopulation OSW (Eastern cluster) appears to be of high concern. Separated by a major valley (the Mur‐Mürz‐Furche), it is the most differentiated subpopulation within the metapopulation, and no immigration from other subpopulations was found. Losing connection to the metapopulation system, subpopulation OSW might end up in reproductive isolation. Given ongoing range contraction through the loss and degradation of habitat (Gehrig‐Fasel et al., 2007; Groier, 2010; Tasser et al., 2007; Theurillat & Guisan, 2001) and increasing disturbance within the remaining habitats (Arlettaz et al., 2007; Coppes et al., 2017; Immitzer et al., 2014; Ingold, 2005), the subpopulations' long‐term survival is therefore threatened (Frankham et al., 2010). Our results might be an early warning signal (Kunz et al., 2021), as extinction events of isolated black grouse populations have been observed in various cases over the past decades (Höglund, 2009; Höglund et al., 2007 and references therein).

Individuals from the Southern cluster seem to be migrating into the Zirbitzkogel and Inneralpine cluster. The subpopulations within the Southern cluster are situated at the administrative border, and it is very likely that they are connected to black grouse populations in Carinthia. Especially the subpopulation KOR might therefore act as an important stepping stone. Surprisingly, no migration was found between the Zirbitzkogel and the Inneralpine cluster. Considering the landscape's permeability, we therefore assume individuals emigrating from the Southern cluster to either settle within Zirbitzkogel or continue dispersing into the Inneralpine cluster. Its role as potential stepping stone for black grouse populations in Carinthia still remains unresolved, and more samples are needed, spanning a wide geographic region. As we only found unidirectional migration, unknown effects might be leading individuals to emigrate and, at the same time, prevent immigration.

### 4.2 Drivers of black grouse spatial genetic variation

We found clear positive significant relationships of genetic differentiation and geographic distances (LCP length and effective resistances) for all our models. On an individual level, analyses resulted in models based on IBD being marginally more explanatory than models based on IBR. The proportion of shared alleles was better explained by the model including the pairwise Euclidean distances among individuals than by the model including the pairwise LCPs across the resistance surface. Pairwise genetic data are known to be noisy, and therefore, inferences are often challenging (Peterman & Pope, 2020). Among individuals, the landscape's resistance arguably did not exert a meaningful effect. The similar performance of the tested models of IBD and IBR might rather indicate a cumulative effect on gene flow, which seems reasonable for a species with restricted dispersal capabilities. Both models were superior to a model based solely on altitude.

On a subpopulation level, the *memgene* analysis was not able to reproduce the patterns found among individuals, which might derive from the fact that *memgene* is working best when genetic distances are more pronounced among individuals than among subpopulations (P. Galpern, pers. comm.). The MLPE models showed the LCP lengths to be the best explaining predictors. Taken together, our results suggest the spatial genetic pattern in the studied black grouse metapopulation system to be driven by IBD among individuals and by IBR effects among subpopulations. Our results did not show distinct differences among models, as shown by the small delta AICc and the proportion of explained variance. Additional factors not represented within our chosen approach might be affecting genetic differentiation beyond geographic distances. We purposely excluded highly variable short‐term environmental factors. Anthropogenic factors and disturbances (e.g., frequencies of hikers and dogs and forestry) might as well exert effects on the spatial genetic variation of black grouse (Arlettaz et al., 2007; Coppes et al., 2017; Immitzer et al., 2014; Ingold, 2005). Studies quantifying these effects for black grouse are still lacking as data of these factors are sparse and mostly not available for larger regions.

Although our results could be taken as indication for the presence of barriers between subpopulations, we assume the observed patterns to be a consequence of unidirectional dispersal and short‐distance dispersal of black grouse. Unidirectional dispersal is common for metapopulation systems experiencing source–sink dynamics (Kawecki, 2004). We found patterns of unidirectional dispersal for several pairs, with especially the outermost subpopulations not receiving alleles from the larger, more central subpopulations. Although dispersal in black grouse is female‐based (Lebigre et al., 2010), no clear evidence has been found for female‐based dispersal affecting black grouse spatial genetic variation (Corrales & Höglund, 2012). Female‐based dispersal rather seems to counteract differentiation effects (Lebigre et al., 2008, 2010). Instead, short‐distance dispersal in general is assumed to lead to a global IBD pattern (overall subpopulations), with potential IBR effects being present at local scales only (Blair et al., 2012). We therefore assume our observed pattern of spatial genetic variation to be a result of short‐distance dispersal. Detection of effects of recent barriers, however, might be difficult, as for short‐distance dispersing species, such effects need several generations to manifest (Landguth et al., 2010). Within our study area, habitat segregation as an ongoing process might be too recent yet to lead to distinct genetic differences. Additionally, a network of remaining patches of suitable habitats between subpopulations serving as stepping stones might have prevented subpopulations from distinct differentiation in the past. In the light of increasing habitat loss and fragmentation, it becomes vital to reassess population structure and connectivity on a regular basis, in order to understand a species' response to landscape features and detect potential barriers for gene flow.

A key component within landscape genetic analyses is the parameterization of the resistance surface. In the past decades, expert‐based resistance surfaces were widely applied to extract measures of geographic distances (Epps et al., 2007; Shirk et al., 2010). More recently, correlative ENMs have increasingly been used due to their continuous and objective nature (Milanesi, Holderegger, Caniglia, et al., 2017; Wang et al., 2008). Although ENMs succeed in identifying habitats of species, they were, however, suspected to inaccurately predict landscape elements that are essential during movement or dispersal (Keller et al., 2013). As an alternative, resistance surfaces produced through optimization approaches were suggested (Mateo‐Sánchez et al., 2015). There is, however, no single optimal approach applicable for all circumstances. Instead, the parameterization of resistance models depends on various factors, including the study objectives and the species' biology (Spear et al., 2010). As black grouse is mainly ground‐dwelling and dispersal is generally low, movement and dispersal behavior are assumed to be driven by habitat structures, especially the availability of food resources and protection (against predators and adverse weather conditions). Accordingly, the resistance of a landscape can be assumed to be reflected by the spatial distribution of suitable areas that offer such resources at finer scales (Milanesi, Holderegger, Caniglia, et al., 2017). We consider black grouse to exhibit back‐and‐forth movements driven by the landscape's suitability (Baguette & Van Dyck, 2007; Van Dyck & Baguette, 2005). We therefore based our resistance model on a validated correlative ENM by using a vast amount of presence data and potential variables, accounting for spatial autocorrelation and multicollinearity and applying stepwise top‐down selection of variables and parameters. This allowed us to model a composite resistance surface prior to extracting distance measures instead of using single environmental variables, as recently recommended (Peterman & Pope, 2020).

Interestingly, effective resistances as circuit theory‐based measurements for IBR were outperformed in all analyses by the cost distance‐based models (LCP lengths) between subpopulations. Dispersal in black grouse most likely happens at an individual's prereproductive stage (Caizergues & Ellison, 2002; Corrales & Höglund, 2012) and is not traditionally passed on over generations. Therefore, one might expect circuit theory‐based approaches to be more suited, as these approaches presume that individuals have no prior knowledge of the landscape apart from their immediate surroundings and incorporate redundancy in pathways between source and destination. Yet, LCP length showed higher explanatory power. We assume this to be due to dispersal between pairs of subpopulations being geographically restricted (by high mountain ridges and valleys densely populated by humans) and therefore often only allowing for one dispersal route, which seemed to be represented by the LCPs.

### 4.3 Consequences for conservation

Black grouse were historically widespread in Europe, ranging from Alpine areas to lowland habitats, yet human landscape alteration within the last centuries in Central Europe resulted in the species to retract to the subalpine tree‐line ecotones (Sachser et al., 2017). Our ENM clearly shows current habitats to be restricted to those areas. The landscape is highly fragmented, with unsuitable areas to some extend exceeding dispersal distances (approximately 8 km; Caizergues & Ellison, 2002; Marjakangas & Kiviniemi, 2005; Warren & Baines, 2002; Willebrand, 1988). Such areas are predominately major valleys of several kilometers widths, characterized by low altitude and high density of anthropogenic settlements and infrastructure or high mountain ridges. Connectivity of subpopulations seems to follow a metapopulation network (Sittenthaler et al., 2018), with corridors alongside the LCPs.

The easternmost occurrences of black grouse in our study area also represent the easternmost Alpine distribution of the species (BirdLife International, 2016) and losses of connectivity in this region might not be compensated, as shown by past extinction events (Wöss & Zeiler, 2003). Despite large valleys representing barriers to connectivity, other barriers like power lines might impede successful dispersal by causing collision mortality (Baines & Andrew, 2003 and references therein). Thus, two major conservation targets should be particularly addressed for this high priority conservation zone: (1) prevention of a further increase of distances between patches of high habitat suitability paired with establishment of potential stepping stones; this includes all management actions, which aim at an improvement or maintenance of high‐quality habitat patches for the target species (e.g., alpine pasturing, no further development for recreational issues, and reduction of human disturbances; Immitzer et al., 2014; Sachser et al., 2017; Schweiger et al., 2012). (2) Removal of any additional barrier effects, for example, deriving from power lines. Our results indicate that habitat management and species conservation actions need to be based on landscape ecological analyses, which have in turn to be translated into landscape planning processes.

## 5 CONCLUSION

For the Alpine black grouse metapopulation system, preservation of gene flow appears as a primary conservation target (Caizergues et al., 2003; Höglund, 2009). Extinction events of several occurrences in the past decades (Wöss & Zeiler, 2003) and recent genetic differentiation (Sittenthaler et al., 2018) highlight the need for improved connectivity between subpopulations (Höglund et al., 2007). Within in‐situ conservation and landscape planning, Euclidean distances between habitats of subpopulations are often considered and compared with average and maximum dispersal distances of the targeted species (Segelbacher & Storch, 2002; van Strien et al., 2015), thereby accounting for IBD. This approach is uncoupled from any underlying landscape characteristics. We showed that IBR effects between local subpopulations should be considered. Therefore, our ENM provides a valuable addition to landscape planning processes. Overall, Alpine black grouse in the Austrian province of Styria, situated at the eastern border of the species' Alpine distribution, exist within a metapopulation system with currently moderate levels of differentiation. However, the easternmost subpopulation OSW, separated from the Inneralpine occurrences by a major valley, shows first signs of isolation and should be monitored with special attention to prevent its extinction in the upcoming years.

## CONFLICT OF INTEREST

The authors declare no conflicts of interest.

## AUTHOR CONTRIBUTIONS

**Florian Kunz:** Conceptualization (equal); formal analysis (equal); writing – original draft (lead); writing – review and editing (lead). **Peter Klinga:** Conceptualization (equal); formal analysis (equal). **Marcia Sittenthaler:** Conceptualization (equal); writing – review and editing (equal). **Martin Schebeck:** Writing – review and editing (equal). **Christian Stauffer:** Writing – review and editing (equal). **Veronika Grünschachner‐Berger:** Funding acquisition (lead). **Klaus Hackländer:** Writing – review and editing (equal). **Ursula Nopp‐Mayr:** Conceptualization (equal); supervision (lead); writing – review and editing (equal).

## Data Availability

Input files for ecological modeling: owned by the government of the federal state Styria, Austria, accessible upon request; digital elevation model accessible under https://www.data.gv.at/. Geographic locations of sampling sites: sensitive information, accessible upon request. STR genotypes: provided on Dryad under https://doi.org/10.5061/dryad.3ffbg79k3.

## APPENDIX A

**TABLE A1:** Pairwise indices of genetic fixation and differentiation among black grouse subpopulations in Styria, rounded to three digits

	$F_{\text{ST}}$	$G_{\text{ST}}$	$G_{\text{ST}}^{\prime }$	$G_{\text{ST}}^{\prime \prime }$	$D_{\text{Jost}}$
AUS vs. LIN	0.017	0.008	0.037	0.044	0.002
AUS vs. HSS	0.016	0.007	0.037	0.044	0.000
AUS vs. HSW	0.022	0.012	0.062	0.073	0.010
AUS vs. TAU	0.001	0.001	0.007	0.008	0.000
AUS vs. OSW	0.039	0.021	**0.100**	**0.119**	0.028
AUS vs. TUR	0.015	0.006	0.034	0.040	0.000
AUS vs. ZIK	0.040	0.022	**0.102**	**0.121**	0.004
AUS vs. GLS	0.007	0.006	0.029	0.034	0.002
AUS vs. KOR	0.032	0.017	0.076	0.091	0.009
LIN vs. HSS	0.026	0.012	0.059	0.070	0.008
LIN vs. HSW	0.037	0.020	0.098	0.116	0.026
LIN vs. TAU	0.005	0.004	0.022	0.026	0.002
LIN vs. OSW	0.026	0.015	0.067	0.081	0.008
LIN vs. TUR	0.063	0.026	0.133	0.156	0.010
LIN vs. ZIK	0.045	0.025	0.110	**0.132**	0.007
LIN vs. GLS	0.035	0.021	**0.102**	0.120	0.045
LIN vs. KOR	**0.083**	**0.043**	**0.188**	**0.221**	0.054
HSS vs. HSW	0.015	0.007	0.040	0.047	0.007
HSS vs. TAU	0.012	0.006	0.034	0.040	0.006
HSS vs. OSW	**0.040**	**0.020**	**0.103**	**0.121**	0.042
HSS vs. TUR	**0.064**	0.030	**0.174**	**0.198**	0.037
HSS vs. ZIK	**0.076**	**0.039**	**0.193**	**0.223**	0.046
HSS vs. GLS	0.012	0.006	0.034	0.040	0.006
HSS vs. KOR	**0.056**	**0.028**	**0.139**	**0.162**	0.027
HSW vs. TAU	**0.021**	**0.011**	**0.059**	0.069	0.028
HSW vs. OSW	**0.041**	**0.021**	**0.106**	**0.124**	**0.047**
HSW vs. TUR	0.041	0.021	0.118	0.136	0.033
HSW vs. ZIK	**0.048**	**0.025**	**0.122**	**0.144**	0.009
HSW vs. GLS	0.010	0.006	0.033	0.039	0.004
HSW vs. KOR	**0.058**	**0.030**	**0.146**	**0.171**	0.059
TAU vs. OSW	**0.030**	**0.016**	**0.079**	**0.093**	**0.047**
TAU vs. TUR	0.023	0.012	0.068	0.079	0.005
TAU vs. ZIK	**0.038**	**0.021**	**0.098**	**0.116**	0.022
TAU vs. GLS	0.011	0.006	0.032	0.037	0.010
TAU vs. KOR	**0.032**	**0.018**	**0.084**	**0.100**	0.011
OSW vs. TUR	**0.103**	**0.053**	**0.270**	**0.306**	**0.094**
OSW vs. ZIK	**0.050**	**0.027**	**0.118**	**0.141**	**0.055**
OSW vs. GLS	**0.028**	**0.015**	**0.072**	**0.085**	**0.036**
OSW vs. KOR	**0.073**	**0.039**	**0.172**	**0.203**	**0.080**
TUR vs. ZIK	**0.094**	**0.049**	**0.237**	**0.273**	**0.124**
TUR vs. GLS	**0.056**	0.030	**0.166**	**0.190**	0.035
TUR vs. KOR	**0.087**	0.043	**0.210**	**0.242**	0.062
ZIK vs. GLS	**0.040**	**0.022**	**0.104**	**0.123**	0.014
ZIK vs. KOR	**0.065**	**0.035**	**0.149**	**0.177**	0.034
GLS vs. KOR	0.010	0.006	0.030	0.036	0.002

Note

Significant values via 95% bias‐corrected confidence intervals indicated in bold.
